# Supplementary material for: Unsolved Issues in Thymic Epithelial Tumour Stage Classification: The Role of Tumour Dimension
Source: Diagnostics (Basel). 2023 Nov 17;13(22):3468. doi: 10.3390/diagnostics13223468 (PMC10670816; doi:10.3390/diagnostics13223468)
Supplement: Supplementary file 1 [file diagnostics-13-03468-s001.zip › diagnostics-2664246-SI/diagnostics-2664246-figures.pdf]

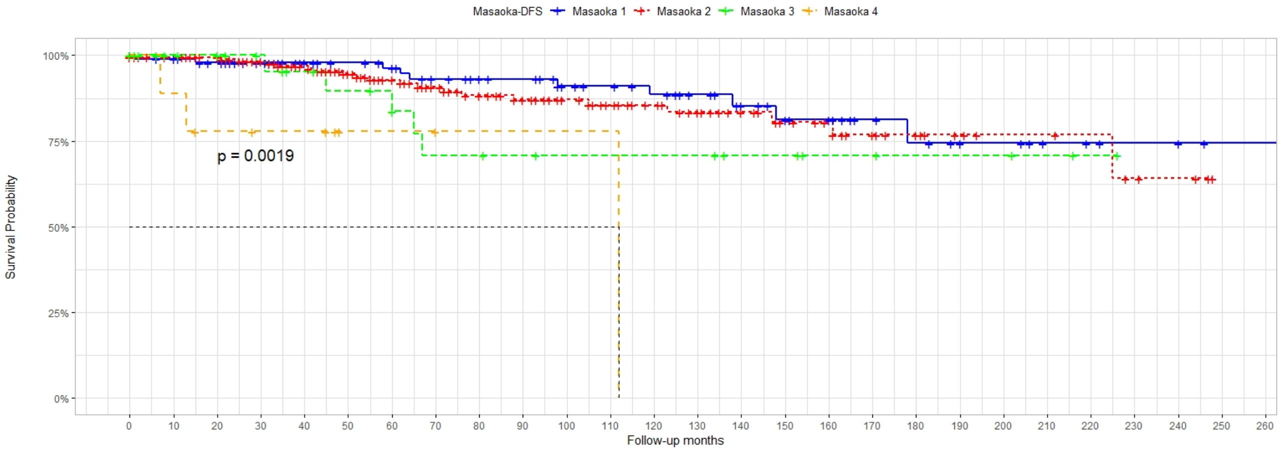


A

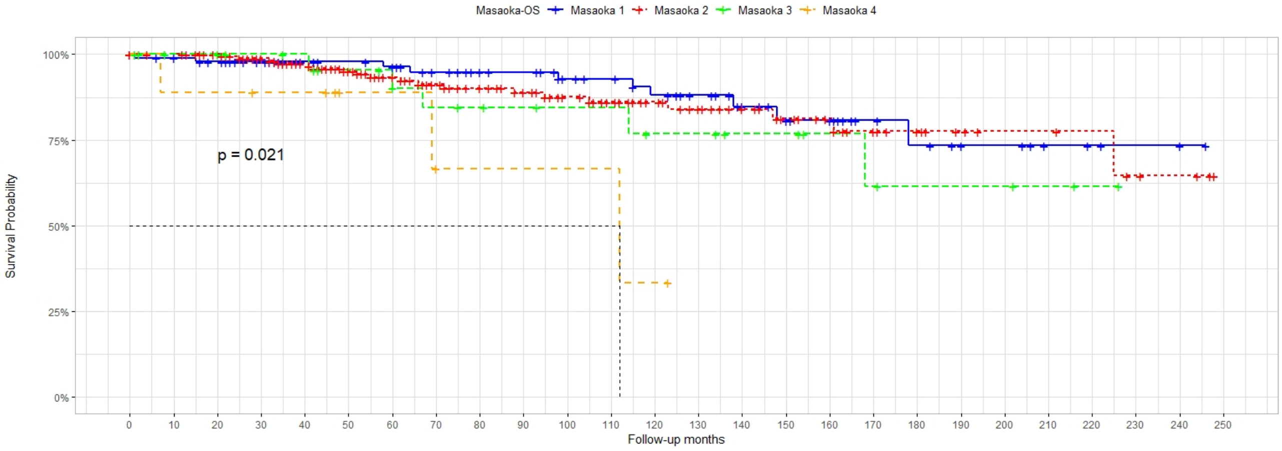


B

**Figure S1** Disease free survival (A) and overall survival (B) according to Masaoka-Koga staging system

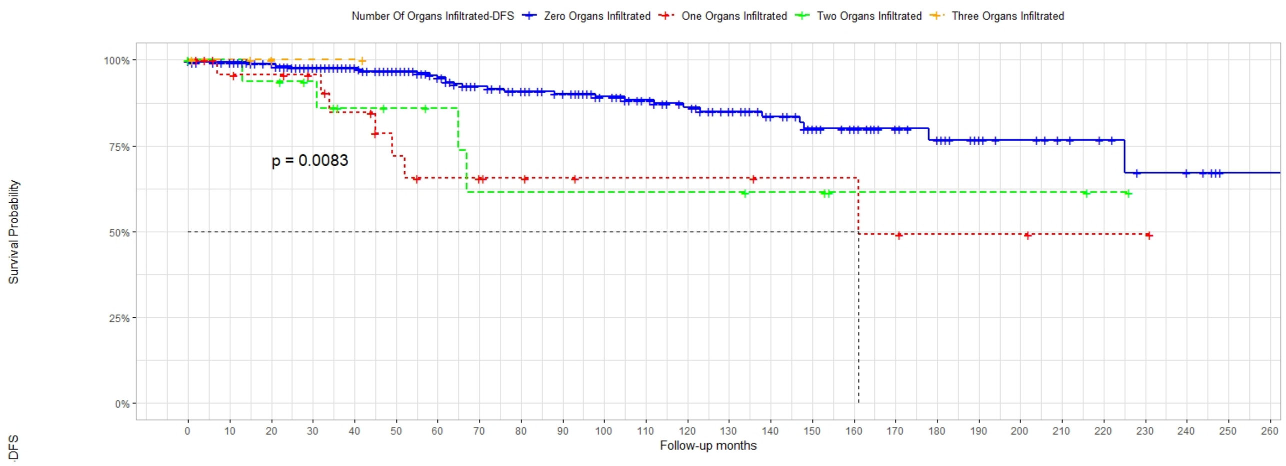


A

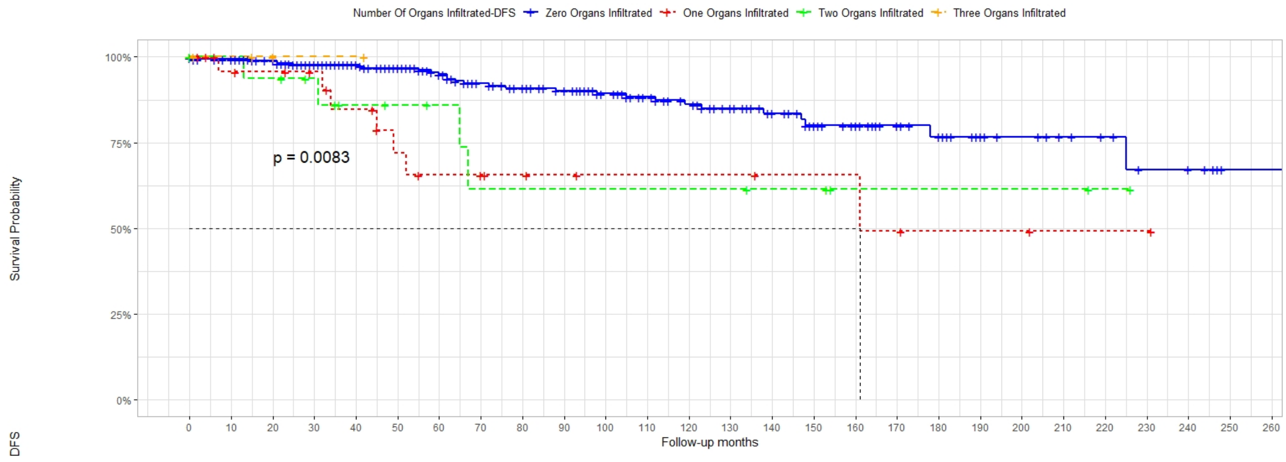


B

**Figure S2** Disease free survival (A) and overall survival (B) according to the number of infiltrated organs

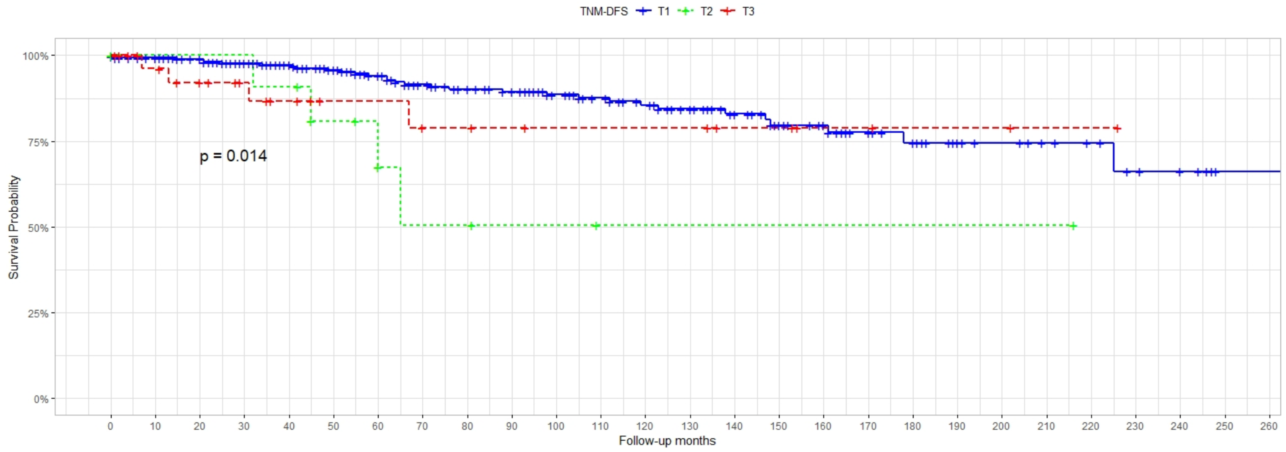


A

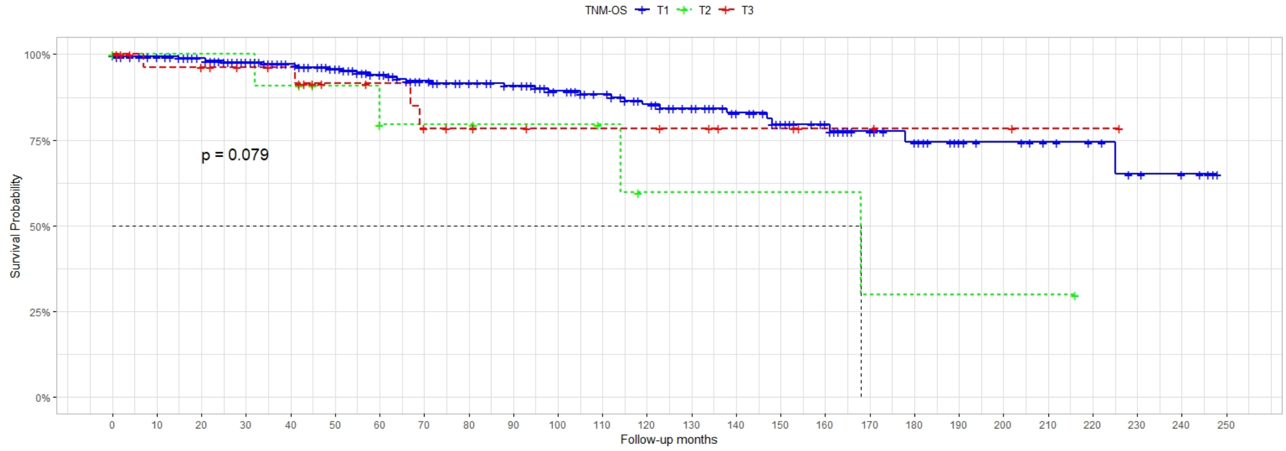


B

**Figure S3** Disease free survival (A) and overall survival (B) according to the TNM staging system
