# Supplementary material for: Unsolved Issues in Thymic Epithelial Tumour Stage Classification: The Role of Tumour Dimension
Source: Diagnostics (Basel). 2023 Nov 17;13(22):3468. doi: 10.3390/diagnostics13223468 (PMC10670816; doi:10.3390/diagnostics13223468)
Supplement: Supplementary file 1 [file diagnostics-13-03468-s001.zip › diagnostics-2664246-SI/diagnostics-2664246-tables.pdf]

N infiltrated organs	Lwr	Upr	p-value	p-value Adj
1-0	-0.36	-1.82	1.13	0.91
2-0	0.89	-0.95	2.74	0.59
**3-0**	3.93	0.21	7.65	**0.03**
2-1	1.25	-1.01	3.53	0.48
**3-1**	4.29	0.33	8.24	**0.02**
3-2	3.03	-1.07	7.14	0.22

Table S1: Tukey HSD analysis for number of infiltrated organs.

	Coef	Exp (Coef)	Lower 95% CI	Upper 95% CI	SE (Coef)	Robust-SE	z-test	Pr(>|z|)
**Continuous variable**	0.04	1.04	0.94	1.15	0.07	0.05	0.83	0.40
**Number of Organs Infiltrated: 1**	1.39	4.04	3.22	5.05	0.78	0.11	12.2	**<0.01**
**Number of Organs Infiltrated: 2**	1.67	5.32	2.66	10.67	0.93	0.35	4.72	**<0.01**
**Number of Organs Infiltrated: 3**	2.43	11.41	8.17	15.93	1.37	0.17	14.29	**<0.01**
**pTNM: pt2**	0.2	1.22	0.16	8.91	1.01	1.01	0.2	0.84
**pTNM: pt3**	-0.26	0.76	0.14	4.1	1.1	0.85	-0.31	0.75
**Masaoka**	0.51	1.67	0.42	6.66	0.45	0.7	0.73	0.46
**cut off 5 cm**	-0.24	0.78	0.29	2.09	0.45	0.5	-0.49	0.62
**Number of Organs Infiltrated: 1**	1.31	3.73	3.46	4.02	0.77	0.03	34.39	**<0.01**
**Number of Organs Infiltrated: 2**	1.53	4.62	1.45	14.75	0.91	0.59	2.58	**0.009**
**Number of Organs Infiltrated: 3**	2.57	13.19	11.85	14.68	1.36	0.05	47.35	**<0.01**
**pTNM: pT2**	0.41	1.5	0.1	21.6	1.01	1.35	0.3	0.76
**pTNM: pT3**	-0.003	0.99	0.08	11.82	1.05	1.26	-0.002	0.99
**Masaoka**	0.48	1.62	0.38	6.76	0.44	0.72	0.66	0.51
**cut off 3 cm**	0.46	1.59	0.77	3.29	0.76	0.36	1.26	0.21
**Number of Organs Infiltrated: 1**	1.38	4	3.64	4.38	0.78	0.04	29.48	**<0.01**
**Number of Organs Infiltrated: 2**	1.62	5.06	2	12.76	0.92	0.47	3.44	**<0.01**
**Number of Organs Infiltrated: 3**	2.53	12.57	11.37	13.9	1.36	0.05	49.39	**<0.01**
**pTNM: pT2**	0.2	1.22	0.14	10.27	1.01	1.08	0.18	0.84
**pTNM: pT3**	-0.18	0.82	0.11	5.79	1.06	0.99	-0.18	0.85
**Masaoka**	0.48	1.62	0.39	6.71	0.44	0.72	0.67	0.51

Table S2: Multivariable model for disease free survival

	Coef	Exp (Coef)	Lower 95% CI	Upper 95% CI	SE (Coef)	Robust-SE	z-test	Pr(>|z|)
**Continuous variable**	0.04	1.04	0.94	1.15	0.07	0.05	0.83	0.40
**Number of Organs Infiltrated: 1**	1.39	4.04	3.22	5.05	0.78	0.11	12.20	**<0.01**
**Number of Organs Infiltrated: 2**	1.67	5.32	2.66	10.67	0.93	0.35	4.72	**<0.01**
**Number of Organs Infiltrated: 3**	2.43	11.41	8.17	15.93	1.37	0.17	14.29	**<0.01**
**PTNM: t2**	0.20	1.22	0.16	8.91	1.01	1.01	0.21	0.84
**PTNM: t3**	-0.26	0.76	0.14	4.10	1.10	0.85	-0.31	0.75
**Masaoka**	0.51	1.67	0.42	6.66	0.45	0.70	0.73	0.46

	Coef	Exp(Coef)	Lower 95% CI	Upper 95% CI	SE (Coef)	Robust-SE	z-test	Pr(>|z|)
**Cut off 5 cm**	-0.29	0.74	0.64	0.86	0.54	0.07	-3.85	**<0.01**
**Number of Organs Infiltrated: 1**	1.57	4.84	3.59	6.53	0.88	0.15	10.33	**<0.01**
**Number of Organs Infiltrated: 2**	1.91	6.78	3.15	14.56	1.02	0.38	4.91	**<0.01**
**Number of Organs Infiltrated: 3**	3.25	25.86	15.51	43.14	1.46	0.26	12.46	**<0.01**
**pTNM: pT2**	0.90	2.46	0.55	10.84	1.21	0.75	1.192	0.23
**pTNM: pT3**	0.63	1.88	1.39	2.55	1.27	0.15	4.101	**<0.01**
**Masaoka**	-0.17	0.84	0.42	1.68	0.56	0.35	-0.481	0.63

	Coef	Exp (Coef)	Lower 95% CI	Upper 95% CI	SE (Coef)	Robust_SE	z-test	Pr(>|z|)
**Cut off 5 cm**	0.91	2.50	0.20	30.58	1.05	1.27	0.72	0.47
**Number of Organs Infiltrated: 1**	1.66	5.25	4.29	6.43	0.88	0.10	16.14	**<0.01**
**Number of Organs Infiltrated: 2**	1.99	7.37	3.61	15.02	1.01	0.36	5.49	**<0.01**
**Number of Organs Infiltrated: 3**	3.19	24.35	14.89	39.81	1.46	0.25	12.73	**<0.01**
**pTNM: pT2**	0.61	1.85	0.58	5.82	1.21	0.58	1.05	0.29
**pTNM: pT3**	0.34	1.40	1.38	1.43	1.27	0.007	42.83	**<0.01**
**Masaoka**	-0.14	0.86	0.42	1.73	0.56	0.35	-0.41	0.68

Table S3: Multivariate model for disease free survival in Thymomas.

	Coef	Exp (Coef)	Lower 95% CI	Upper 95% CI	SE (Coef)	Robust-SE	z-test	Pr(>|z|)
**Continuous variable**	-1.01e^{-01}	9.04e^{-01}	8.44e^{-01}	9.67e^{-01}	7.21e^{-02}	3.48e^{-2}	-2.91	<0.01
**Number of Organs Infiltrated: 1**	9.73e^{-01}	2.65E	1.37	5.12	5.43e^{-01}	3.37e^{-01}	2.88	<0.01
**Number of Organs Infiltrated: 2**	3.13e^{-01}	1.37	7.93e^{-01}	2.36	7.41e^{-01}	2.78e^{-01}	1.12	0.25
**Number of Organs Infiltrated: 3**	-15.10	2.65e^{-07}	5.98e^{-08}	1.18e^{-06}	4.14e^{+03}	7.60e^{-01}	-19.91	<0.01
**Masaoka**	2.10e^{-01}	1.23	5.74e^{-01}	2.65	3.07e^{-01}	3.91e^{-01}	0.53	0.59

	Coef	Exp (Coef)	Lower 95% CI	Upper 95% CI	SE (Coef)	Robust-SE	z-test	Pr(>|z|)
**Cut off 5 cm**	-1.44e^{-01}	8.66e^{-01}	8.11e^{-01}	9.24e^{-01}	3.45e^{-01}	3.32e^{-02}	-4.33	**<0.01**
**Number of Organs Infiltrated: 1**	9.79e^{-01}	2.66	1.34	5.31	5.51e^{-01}	3.52e^{-02}	2.78	**<0.01**
**Number of Organs Infiltrated: 2**	3.12e^{-01}	1.37	7.60e^{-01}	2.46	7.44e^{-01}	2.99e^{-01}	1.04	0.29
**Number of Organs Infiltrated: 3**	-15.40	2.09e^{-07}	4.40e^{-08}	9.97e^{-07}	4.15e^{+03}	7.96e^{-01}	-19.31	**<0.01**
**Masaoka**	1.82e^{-01}	1.20	5.58e^{-01}	2.58	3.10e^{-01}	3.90e^{-01}	0.46	0.64

	Coef	Exp (Coef)	Lower 95% CI	Upper 95% CI	SE (Coef)	Robust SE	z-test	Pr(>|z|)
**Cut off 3 cm**	-6.28e^{-01}	5.34e^{-01}	5.29e^{-01}	5.39e^{-01}	4.03E-01	4.81e^{-03}	-130.63	**<0.01**
**Number of Organs Infiltrated: 1**	8.47e^{-01}	2.33	1.19	4.59	5.55E-01	3.45e^{-01}	2.45	**0.01**
**Number of Organs Infiltrated: 2**	2.98e^{-01}	1.35	7.26e^{-01}	2.50	7.53E-01	3.16e^{-01}	0.94	0.34
**Number of Organs Infiltrated: 3**	-1.55	1.84e^{-07}	3.83e^{-08}	8.83e^{-07}	4.20E+03	8.01e^{-01}	-19.36	**<0.01**
**Masaoka**	2.56e^{-01}	1.29	6.09e^{-01}	2.74	3.18E-01	3.84e^{-01}	0.66	0.51

Table S4: Multivariate model for overall survival in Thymomas.

	Coef	Exp (Coef)	Lower 95% CI	Upper 95% CI	SE (Coef)	Robust-SE	z-test	Pr(>|z|)
**Continuous variable**	-1.37 e^{-01}	8.71 e^{-01}	7.53 e^{-01}	1.00	1.31 e^{-01}	7.44 e^{-02}	-1.84	0.06
**Number of Organs Infiltrated: 1**	1.62 e^{+01}	9.21e^{-08}	2.21 e^{-08}	3.82 e^{-07}	2.08 e^{+04}	7.26 e^{-01}	-22.29	**<0.01**
**Number of Organs Infiltrated: 2**	-1.61 e^{+01}	1.05 e^{-07}	1.60 e^{-08}	6.89 e^{-07}	1.33 e^{+04}	9.59 e^{-01}	-16.74	**<0.01**
**cut-off 5 cm**	-1.92 e^{-01}	8.25 e^{-01}	3.73 e^{-01}	1.82	6.54 e^{-01}	4.04 e^{-01}	-0.47	0.63
**Number of Organs Infiltrated: 1**	-1.61 e^{+01}	9.92 e^{-08}	2.47 e^{-08}	3.97 e^{-07}	2.04 e^{+04}	7.07 e^{-01}	-22.78	**<0.01**
**Number of Organs Infiltrated: 2**	-1.61 e^{+01}	9.96 e^{-08}	1.94 e^{-08}	5.10 e^{-07}	1.31 e^{+04}	8.33 e^{-01}	-19.34	**<0.01**
**cut-off 3 cm**	-6.14 e^{-01}	5.41 e^{-01}	2.35 e^{-01}	1.24	7.01 e^{-01}	4.24 e^{-01}	-1.44	0.14
**Number of Organs Infiltrated: 1**	-1.48 e^{+01}	3.40 e^{-07}	7.07 e^{-08}	1.63 e^{-06}	1.27 e^{+04}	8.01 e^{-01}	-18.58	**<0.01**
**Number of Organs Infiltrated: 2**	-1.49 e^{+01}	3.37 e^{-07}	4.33 e^{-08}	2.62 e^{-06}	8.11 e^{+03}	1.04	-14.24	**<0.01**

Table S5: Multivariate model for overall survival in Masaoka I.
